# Supplementary material for: Antisense Oligonucleotide-Mediated Removal of the Polyglutamine Repeat in Spinocerebellar Ataxia Type 3 Mice
Source: Mol Ther Nucleic Acids. 2017 Jun 29;8:232–42. doi: 10.1016/j.omtn.2017.06.019 (PMC5504086; doi:10.1016/j.omtn.2017.06.019)
Supplement: Document S1. Figures S1–S3 [file mmc1.pdf]

OMTN, Volume *8*

Supplemental Information

Antisense Oligonucleotide-Mediated Removal of the Polyglutamine Repeat in Spinocerebellar Ataxia Type 3 Mice

Lodewijk J.A. Toonen, Frank Rigo, Haico van Attikum, and Willeke M.C. van Roon-Mom

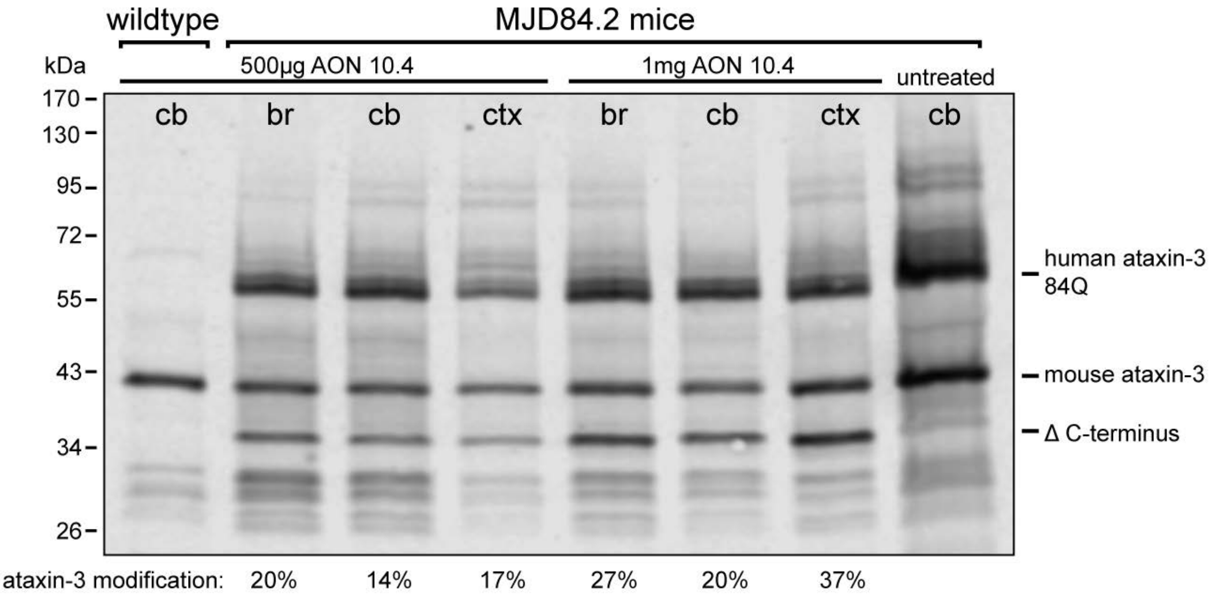

Supplementary Figure 1: Comparison of 500 µg and 1 mg AON 10.4 ICV bolus. AON 10.4 was tested *in vivo* by ICV injection in two mice to determine dose response. 500 µg AON was injected at 2.5 months of age, after which one of the mice was injected with a second 500 µg bolus 2 weeks later. Both mice were sacrificed 2 weeks after the last injection. Westernblot analysis and staining with 1H9 antibody shows ataxin-3 Δ C-terminus appearing in the three brainregions tested of the treated MJD84.2 mice. A clear increase in protein modifciation is seen with the 1 mg dose compared to the 500 µg dose. Cb = cerebellum, br = brainstem, ctx = cortex.

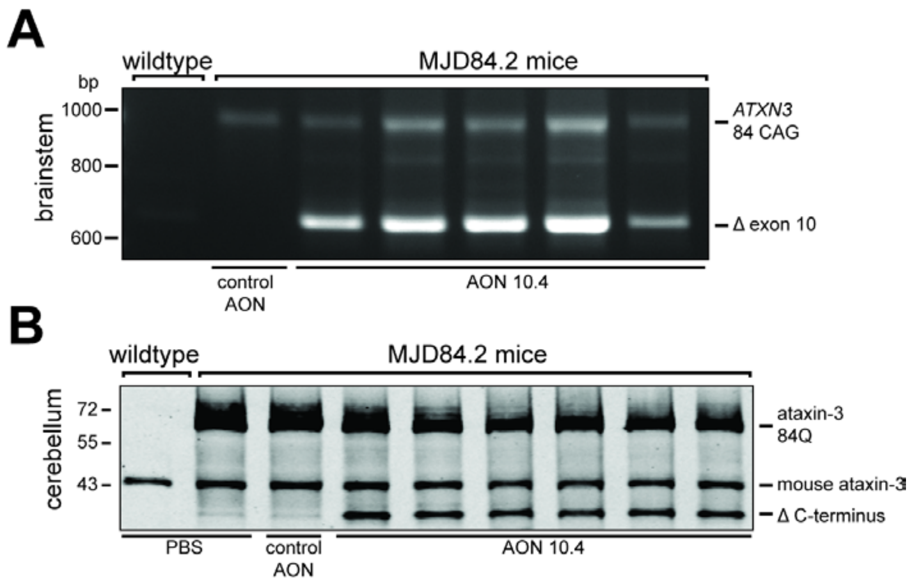

Supplementary Figure 2: In vivo assessment of exon skipping at RNA and protein level. Mice were treated with a total of 1 mg AON 10.4 or control AON, and sacrificed ~3.5 monhs after last injection. Results are obtained from mice depicted in figure 3. (**A**) RT-PCR with primers for human *ATXN3* show skipping of exon 10 in brainstem of AON 10.4 treated mice. (**B)** Modified ataxin-3 protein (Δ C-terminus) was observed in cerebellum of mice treated with AON 10.4.

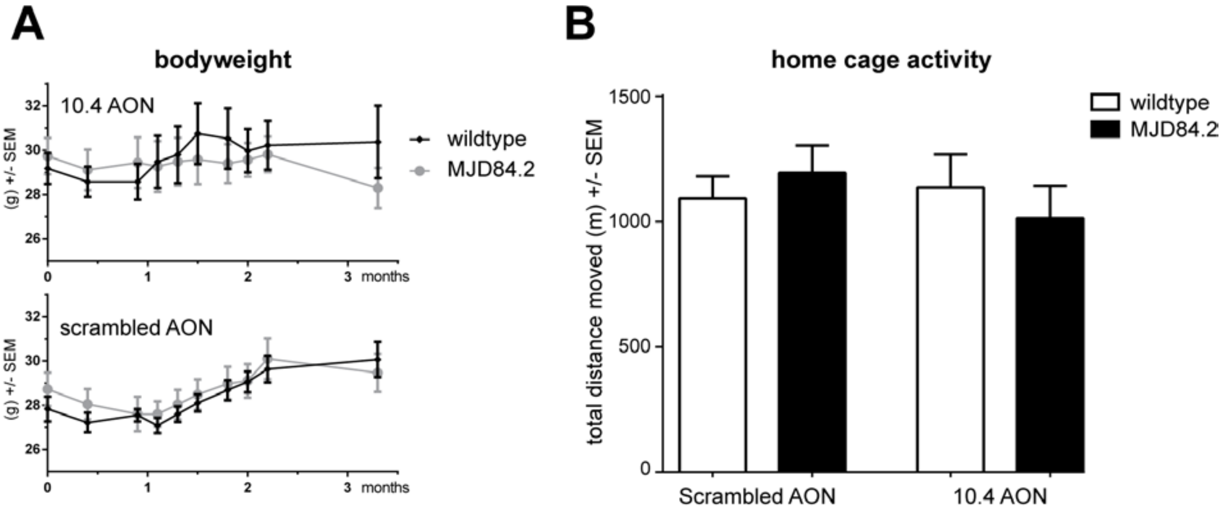

Supplementary Figure 3: MJD84.2 mouse does not present obvious ataxic phenotype at 5 months of age. (A) No significant difference in bodyweight between wildtype and transgenic mice was seen, or between scrambled and AON10.4 treated mice of both genotypes. (**B**) Wildtype and hemizygous MJD84.2 mice were tested for motor performance in a home cage activity system at around 5 months of age. Scrambled AON: 5 wildtype vs 5 SCA3 mice. 10.4 AON: 4 wildtype vs 6 SCA3 mice.
