# Supplementary material for: Antisense Oligonucleotide-Mediated Removal of the Polyglutamine Repeat in Spinocerebellar Ataxia Type 3 Mice
Source: Mol Ther Nucleic Acids. 2017 Jun 29;8:232–42. doi: 10.1016/j.omtn.2017.06.019 (PMC5504086; doi:10.1016/j.omtn.2017.06.019)
Supplement: Document S2. Article plus Supplemental Information [file mmc2.pdf]

Molecular Therapy
Nucleic Acids
Original Article

AMERICAN SOCIETY of GENE & CELL THERAPY

Antisense Oligonucleotide-Mediated Removal of the Polyglutamine Repeat in Spinocerebellar Ataxia Type 3 Mice

Lodewijk J.A. Toonen,[1] Frank Rigo,[2] Haico van Attikum,[1] and Willeke M.C. van Roon-Mom[1]

[1]Department of Human Genetics, Leiden University Medical Center, Albinusdreef 2, 2333ZA Leiden, the Netherlands; [2]Ionis Pharmaceuticals, Carlsbad, CA 92008, USA

Spinocerebellar ataxia type 3 (SCA3) is a currently incurable neurodegenerative disorder caused by a CAG triplet expansion in exon 10 of the *ATXN3* gene. The resultant expanded polyglutamine stretch in the mutant ataxin-3 protein causes a gain of toxic function, which eventually leads to neurodegeneration. One important function of ataxin-3 is its involvement in the proteasomal protein degradation pathway, and long-term downregulation of the protein may therefore not be desirable. In the current study, we made use of antisense oligonucleotides to mask predicted exonic splicing signals, resulting in exon 10 skipping from *ATXN3* pre-mRNA. This led to formation of a truncated ataxin-3 protein lacking the toxic polyglutamine expansion, but retaining its ubiquitin binding and cleavage function. Repeated intracerebroventricular injections of the antisense oligonucleotides in a SCA3 mouse model led to exon skipping and formation of the modified ataxin-3 protein throughout the mouse brain. Exon skipping was long lasting, with the modified protein being detectable for at least 2.5 months after antisense oligonucleotide injection. A reduction in insoluble ataxin-3 and nuclear accumulation was observed following antisense oligonucleotide treatment, indicating a beneficial effect on pathogenicity. Together, these data suggest that exon 10 skipping is a promising therapeutic approach for SCA3.

INTRODUCTION

Spinocerebellar ataxia type 3 (SCA3) is a hereditary neurodegenerative disorder characterized by ataxia, usually presenting in the third to sixth decade of life.[1] Pathoanatomical studies of SCA3 patient brains have shown neurodegeneration in the cerebellum, thalamus, midbrain, pons, medulla, and spinal cord.[2] SCA3 is one of nine known polyglutamine (polyQ) disorders. The causative mutation for polyQ disorders is a CAG codon repeat expansion in the coding region of a gene, which upon translation leads to an expanded glutamine amino acid stretch in the causative protein. It is thought that the polyQ expansion leads to a gain of toxic protein function.[3] In the case of SCA3, the CAG repeat expansion is located in exon 10 of the *ATXN3* gene, encoding the ataxin-3 protein.[4] In the normal population, an *ATXN3* CAG repeat length of 10 to 51 repeats is observed, while SCA3 patients have repeat lengths of 55 or longer.[5]

Ataxin-3 is a ubiquitously expressed deubiquitinating enzyme of around 42 kDa, with its main function in the proteasomal protein degradation pathway.[6] Several ataxin-3 isoforms have been described,[7] with the predominant ataxin-3 isoform (NCBI: NM_004993) in human and mouse brain containing three ubiquitin-interaction motifs (UIMs).[8,9] It has been shown that the interaction between ataxin-3 and K48-linked poly-ubiquitin chains is dependent on the first two UIMs, while the third UIM located C-terminally of the polyQ stretch appears dispensable for this process.[10,11] Upon binding of poly-ubiquitin chains by the UIMs, the N-terminal catalytic Josephin domain of ataxin-3 can cleave these chains. Through this process, ataxin-3 can facilitate substrate entry into the proteasome, as well as mediate other ubiquitin-dependent pathways.[11,12]

Given the monogenetic nature, SCA3 is an ideal candidate for therapies that specifically target the *ATXN3* gene product. Indeed, several RNAi strategies that downregulate ataxin-3 protein expression have been investigated over the last decade. Non-allele-specific silencing of ataxin-3 was found to reduce neuropathology in a SCA3 rat model.[13] Additionally, by making use of a SNP associated with the mutant *ATXN3* allele (rs12895357), an allele-specific RNAi-mediated reduction of mutant ataxin-3 decreased neuropathological abnormalities and/or motor deficits in both SCA3 rats and mice.[14–16] Antisense oligonucleotides (AONs) are another tool under investigation for therapeutic intervention in SCA3. Particularly for use in the brain, AONs offer several favorable properties, including good distribution throughout the brain after infusion in the cerebrospinal fluid, excellent uptake by neurons and other brain cells, high stability with a half-life of several months, and a promising tolerability in clinical trials thus far.[17] Another advantage of using AONs is that they can also be used to redirect splicing by including or excluding specific exons.

Here, we describe an AON-based strategy to redirect splicing of the ataxin-3 pre-mRNA. The aim of this approach is to remove the toxic

Received 2 February 2017; accepted 24 June 2017;
http://dx.doi.org/10.1016/j.omtn.2017.06.019.

Correspondence: Willeke M.C. van Roon-Mom, Department of Human Genetics, Leiden University Medical Center, Albinusdreef 2, 2333ZA Leiden, the Netherlands.
E-mail: w.vanroon@lumc.nl

 © 2017 The Author(s).
This is an open access article under the CC BY license (http://creativecommons.org/licenses/by/4.0/).

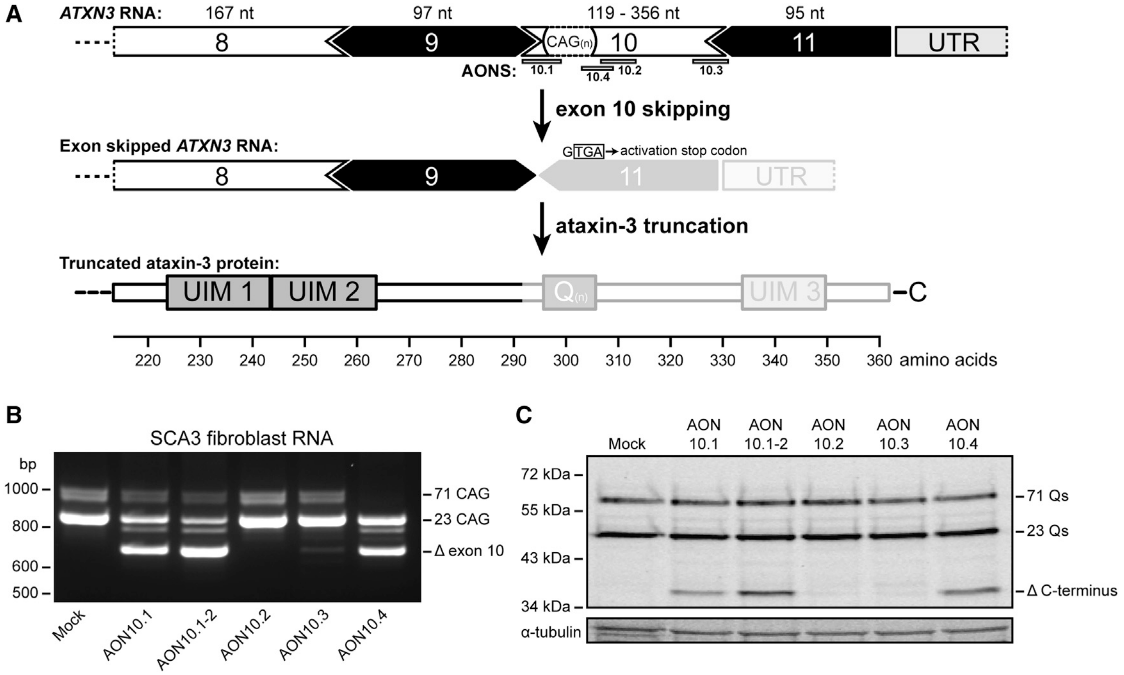

Figure 1. Antisense Oligonucleotide-Mediated Removal of the Polyglutamine Repeat from the Ataxin-3 Protein

(A) Schematic representation of the experimental approach to remove exon 10 from the ataxin-3 mRNA approach based on transcript ENST00000558190 (361 amino acids) using antisense oligonucleotides (AONs) targeting predicted splicing motifs within exon 10. Skipping of exon 10 leads to a novel stop codon at the start of exon 11. The resulting ataxin-3 protein contains 291 amino acids and lacks the polyQ repeat and C terminus (depicted as transparent region). Five different AONs against exon 10 were tested for functionality by transfection in SCA3 patient-derived fibroblasts. (B and C) An internally truncated ataxin-3 RNA lacking the CAG repeat (Δ exon 10) (B) and a truncated ataxin-3 protein lacking the polyQ-containing C terminus (Δ C terminus) (C) were detected. Nt = nucleotides.

polyQ repeat from the mutant ataxin-3 protein, thereby removing the cause of SCA3. Using an AON targeting exon 10 of *ATXN3*, this exon is removed from the pre-mRNA, resulting in a truncated ataxin-3 protein consisting of 291 amino acids with a predicted mass of 34 kDa. Functional testing of the truncated protein lacking the polyQ domain and the third UIM showed that this modified ataxin-3 can still bind ubiquitin chains similar to wild-type ataxin-3. Finally, the AON was tested in the MJD84.2 SCA3 mouse model using repeated bolus injection in the lateral ventricle of the brain. Remarkably, this led to widespread distribution of the AON with ataxin-3 protein modification observed throughout the brain.

RESULTS

Exon 10 Skip Leads to a Truncated Ataxin-3 Protein Lacking the PolyQ Repeat

Removal of exon 10 from the ataxin-3 pre-mRNA results in a stop codon right at the start of exon 11. As a consequence, the resulting ataxin-3 protein is truncated to amino acid 291 (Figure 1A) and lacks the C-terminal region that contains the toxic polyQ repeat, as well as UIM 3. A total of five splice modulating AONs were designed targeting exon 10 (Table 1) and were transfected in SCA3 patient-derived fibroblasts. RT-PCR and western blot analysis showed that three of the tested AONs induced exon 10 skipping as both expression of a shorter ataxin-3 transcript (Δ exon 10) and truncated protein (Δ C terminus) were observed (Figures 1B and 1C). Although AON 10.4 contained an SNP (rs12895357) associated with the expanded allele,[18,19] we always observed modification of both wild-type and mutant alleles.

Ataxin-3 Lacking PolyQ Binds and Cleaves Ubiquitin Chains Similar to Wild-Type

Due to the novel stop codon in exon 11 induced by exon 10 skipping, the ataxin-3 Δ C terminus protein lacks the third UIM (Figure 1A). To determine whether ataxin-3 Δ C terminus was still capable of binding ubiquitin chains, we made use of a U2OS 2-6-3 cell line containing a large array of LacO repeats,[20] in which expression of mCherry-LacR-RNF8 fusion protein leads to localized ubiquitilation of the chromatin.[21] Ataxin-3 subsequently binds the ubiquitins as mediated by the UIMs, resulting in colocalization between mCherry-RNF8 and GFP-ataxin-3. We have previously shown this colocalization of ataxin-3 to the LacO array as a reliable marker for ubiquitin binding activity of ataxin-3.[22] Here, we tested several GFP-tagged ataxin-3 proteins for ubiquitin binding capacity (Figure 2A). In line with previous observations, the ataxin-3 with either 10Q or 71Q can readily bind ubiquitin chains and hence colocalized with the ubiquitin moieties at the array (Figures 2B and 2C). Ataxin-3 with all three UIMs inactivated by point mutations (L229A, L249A, and L340A), termed UIMs L > A, colocalized with the ubiquitin conjugates to a significantly lower extent, confirming the specificity of the

Table 1. *ATXN3* Exon 10 Antisense Oligonucleotides

AON Name	Sequence (5′ to 3′)
10.1	GCTGTTGCTGCTTTTGCTGCTG
10.1-2	CTGTTGCTGCTTTTGCTGCT
10.2	GAACTCTGTCCTGATAGGTC
10.3	CTAGATCACTCCCAAGTGCT
10.4	ATAGGTCCCGCTGCTGCT

assay. Ataxin-3 Δ C terminus colocalized with the ubiquitin conjugates at the array to a similar extent as wild-type ataxin-3, indicating that the ubiquitin chain binding capacity of the protein was retained despite lacking the third UIM.

To confirm the ubiquitin cleavage capacity of ataxin-3 Δ C terminus, purified HIS-ataxin-3 proteins (Figure 2D) were incubated with K63-linked hexa-ubiquitin chains. Similar to ataxin-3 10Q and 71Q, ataxin-3 Δ C terminus was able to cleave ubiquitin chains as evidenced by the appearance of shorter ubiquitin fragments (penta- and tetra-ubiquitin) (Figure 2E). In line with previous reports, deletion of a cysteine in the Josephin domain (ΔC14) abolished ataxin-3 ubiquitin protease activity.[23] Together, these results indicate that removal of the C terminus from ataxin-3 does not interfere with ubiquitin binding and cleavage capacity.

Ataxin-3 Exon Skipping in Mouse Brain

To determine whether the ataxin-3 exon skip strategy is feasible in vivo, the four AONs against *ATXN3* RNA were tested in the MJD84.2 mouse model.[24] The MJD84.2 mouse contains the full human *ATXN3* gene with 84 CAGs including introns and flanking regions, making it a suitable SCA3 rodent model to assess human *ATXN3* splicing events in vivo. Sequencing analysis showed that the human *ATXN3* gene in this mouse also contains the SNP (rs12895357) and thus has full complementarity with AON 10.4 (data not shown). To assess AON efficacy, the AONs targeting exon 10 were injected as a single 500 μg intracerebroventricular (ICV) bolus in anesthetized hemizygous MJD84.2 mice. At 2 weeks after the injection, exon skipping was analyzed in the cortex and cerebellum (Figure 3A). AON efficacy in both brain regions was similar to that observed in SCA3 fibroblasts transfections, with AON 10.4 being most efficient. Sanger sequencing confirmed that the shorter *ATXN3* RNA was the result of exon 10 skipping in the human transcript. In line with the transcript modification, a modified Δ C terminus ataxin-3 protein of approximately 36 kDa in size was observed in the treated animals (Figure 3B). Protein modification appeared more efficient in the cortex than cerebellum. In the untreated animals, an ataxin-3 protein of similar size as Δ C terminus ataxin-3 was observed as well, perhaps indicating the truncated ataxin-3 protein is a naturally occurring isoform or cleavage fragment in these mice.

AON Effect Lasts at Least 2.5 Months in Mouse Brain

Despite relatively high expression of mutant ataxin-3 throughout the brain, hemizygous MJD84.2 mice do not develop an ataxic phenotype on beamwalk tests,[25] but do develop a neuronal phenotype consisting of nuclear localization of mutant human ataxin-3 in certain neuronal populations from around 2 months of age.[25,26] To assess long-term efficacy of AONs in the mouse brain and the effect on the neuronal phenotype, a total of 27 mice were treated with either PBS, scrambled control AON or AON 10.4, and sacrificed 2.5 months after the last injection. With repeated injections, a total ICV dose of 1 mg AON was achieved over a 3 week period (Figure 4A). There was a clear dose-dependent effect on protein modification, with a 500 μg dose resulting in 17% protein modification in the cortex and 1 mg AON resulting in 37% protein modification (Figure S1). At the time of sacrifice, *ATXN3* exon 10 skipping was seen at RNA level in all tested brain regions (Figures 4B and S2A), 40% ataxin-3 modification was seen in protein lysates from the brainstem and cortex, while 20% ataxin-3 modification was seen in the cerebellum (Figures 4C, 4D, and S2B). This indicates that the AONs have distributed throughout the brain and are stable and effective for at least 2.5 months post injection. No deleterious effect of the AON on bodyweight or motor behavior (Figure S3) was observed during the course of the study.

AON Treatment Reduces Insoluble Ataxin-3 and Prevents Nuclear Accumulation

Protein aggregation is considered a hallmark of polyQ disorders. It has been implicated as a pathogenic mechanism for these diseases,[27,28] and mutant ataxin-3 aggregates are found in the brain of SCA3 patients.[29] Using protein lysates from three different brain regions, we performed filter trap assays[30] to detect the level of insoluble ataxin-3 proteins (Figure 4F). Insoluble ataxin-3 protein was detected in the transgenic MJD84.2 mice expressing mutant ataxin-3, but not in wild-type mice. The highest level of insoluble ataxin-3 was detected in the cerebellum. No significant difference in ataxin-3 insolubility was observed between scrambled AON- and PBS-treated SCA3 animals. Following AON 10.4 treatment, there was significantly less insoluble ataxin-3 detected compared to animals treated with scrambled AON or PBS (Figure 4G).

To further assess the effect of AON treatment on expanded ataxin-3 pathogenicity, we performed immunofluorescent examination of SCA3 mouse brains in mice that were ~5.5 months of age at time of sacrifice. We observed strong nuclear ataxin-3 staining throughout the brainstem, but specifically in the substantia nigra. Nuclear accumulation of expanded ataxin-3 has been shown to aggravate neurodegeneration and formation of aggregates in vivo[31] and is therefore a useful marker to assess ataxin-3 toxicity. In fact, nuclear localization of ataxin-3 appears to be required to induce symptoms of SCA3.[31] In the MJD84.2 mouse model, we observed strong nuclear localization of ataxin-3 in the substantia nigra. Therefore, we used tyrosine hydroxylase staining to delineate the substantia nigra in sagittal brain sections (Figure 5A). The intensity of ataxin-3 nuclear localization in the substantia nigra was markedly reduced in mice treated with 10.4 AON when compared to animals treated with control AON (Figure 5B).

DISCUSSION

In this work, we describe an AON-mediated exon skipping strategy for SCA3. Our therapeutic approach removes the polyQ repeat

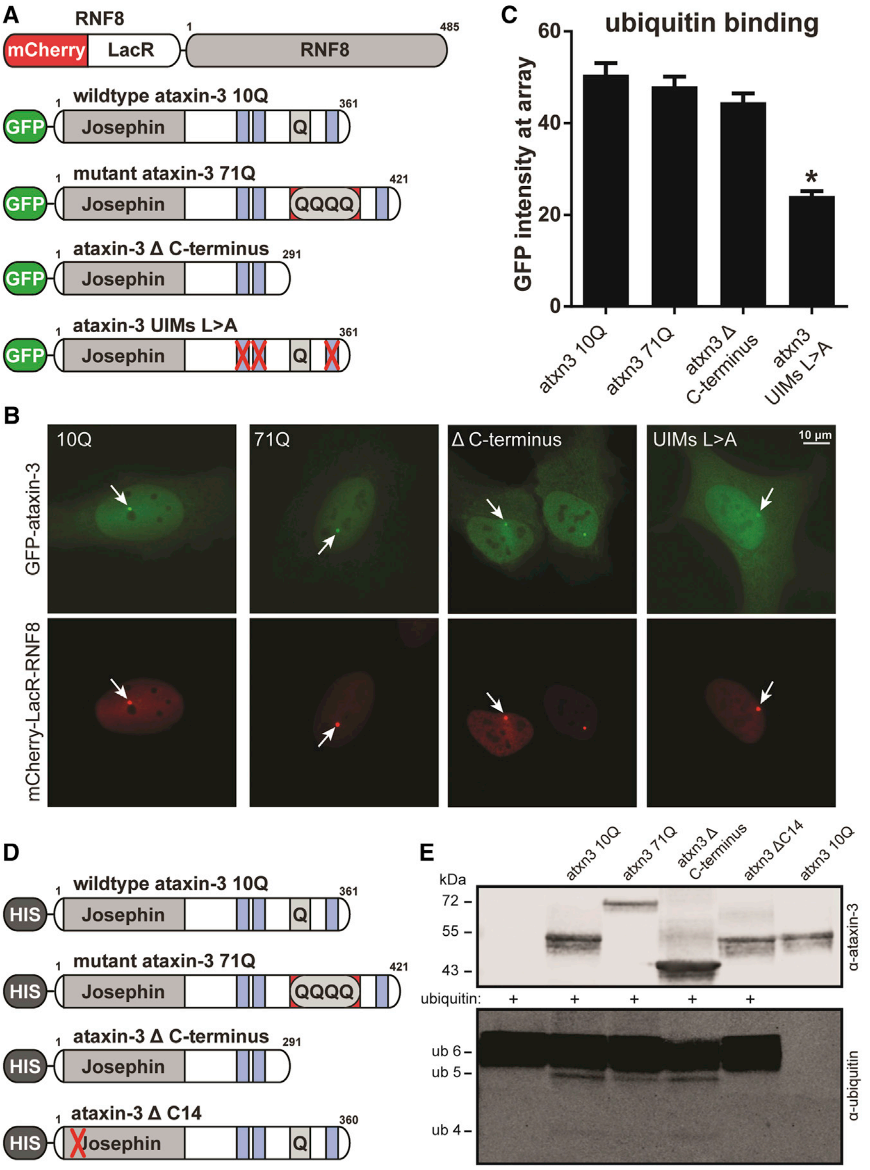

Figure 2. Ataxin-3 Δ C Terminus Is Capable of Binding and Cleaving Ubiquitin Chains

(A) Schematic representation of mCherry and GFP fusion proteins with their functional domains. The ubiquitin ligase RNF8 was fused to mCherry and LacR. Atatxin-3 was fused to GFP. Ubiquitin-interacting motifs (UIMs) of ataxin-3 are depicted in blue. Inactivating substitution mutations are indicated by red crosses. (B) Tethering of the mCherry-LacR-RNF8 fusion protein to a LacO array in the chromatin in U2OS 2-6-3 cells (red signal) results in local chromatin ubiquitylation at the LacO array.[21] GFP-ataxin-3 proteins bind the RNF8-induced ubiquitin moieties at the LacO array, thereby colocalizing with mCherry-LacR-RNF8 (green).[22] Representative images are shown in the panel. The arrows indicate the chromatin localized RNF8 and ataxin-3 where GFP intensity was quantified. (C) Quantification of increase in GFP-tagged ataxin-3 signal at the array after coexpression with mCherry-LacR-RNF8. The values represent the mean + SEM of >40 cells examined from two independent experiments. (D) Schematic representation of HIS-tagged ataxin-3 proteins used in ubiquitin cleavage reaction. The C14 deletion (ΔC14) is known to inactivate catalytic activity. (E) Purified HIS-ataxin-3 proteins were incubated for 16 hr with K63-linked hexa-ubiquitin to determine ubiquitin cleavage activity.

from the protein and therefore removes the cause of SCA3. This approach is superior to current treatments that only treat symptoms and not the cause of disease. It may also have some specific advantages over strategies that aim to lower expression of the ataxin-3 protein, since ataxin-3 has important cellular functions[32] in regulating protein degradation,[23] transcription,[33] and DNA damage response.[34] Though ataxin-3 knockout appears tolerated in mice without major deficits,[35–37] it is currently unknown whether long-term downregulation will be tolerated in patients. Therefore, it is more prudent to avoid complete ataxin-3 downregulation. We provide evidence that repeated ICV injections of AONs in a transgenic SCA3 mouse model leads to substantial ataxin-3 RNA and protein modification (ataxin-3 Δ C terminus) throughout the mouse brain and is able to alleviate ataxin-3 nuclear accumulation and insolubility.

Ataxin-3 Δ C terminus still contains the main known functional domains, namely, the catalytic Josephin domain (amino acids 1–180) and the valosin-containing protein (VCP)-interacting motif (amino acids 257–291).[38] The Josephin domain is required for ataxin-3 deubiquitinating activity,[39] in conjunction with UIM 1 and 2 coordinating specific ubiquitin chain cleavage.[11,12] The UIMs are required for the ubiquin binding capacity of ataxin-3, but the third UIM is not considered essential for this process.[10,11,40,41] The current study confirms this by showing the wild-type ubiquitin binding capacity of Δ C terminus ataxin-3 in a cellular context (Figures 2B and 2C). Previous experiments have shown that removing the C terminus from ataxin-3 does not impair its deubiquitinating activity.[42] Our results are in line with these previous studies and show that ataxin-3 Δ C terminus is capable of cleaving ubiquitin chains (Figure 2E).

Ataxin-3 interaction with VCP is thought to be crucial for the activation of ataxin-3 and regulating the endoplasmic reticulum-associated degradation pathway through protein extraction from the endoplasmic reticulum.[43,44] Ataxin-3 Δ C terminus still contains the VCP binding motif.[38] Retainment of the VCP binding site in ataxin-3 Δ C terminus may be of particular importance, as a mouse model expressing only the ataxin-3 N terminus up to amino acid 259 resulted in extranuclear neuronal aggregates and premature death

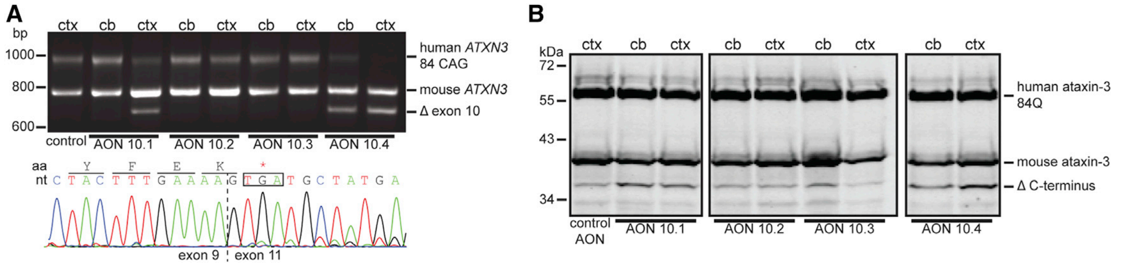

Figure 3. AON Screening In Vivo

500 μg AONs were injected intracerebroventricularly in MJD84.2 mice. After 2 weeks, mice were sacrificed and RNA and protein were isolated from the cortex and cerebellum. (A) RT-PCR using primers flanking *ATXN3* exon 10 shows the full-length human ataxin-3 PCR product with 84 CAG repeats, the mouse ataxin-3 with six CAG repeats, as well as the shorter ataxin-3 lacking exon 10 after injection of AON 10.1 and 10.4 (Δ exon 10). Sanger sequencing trace of Δ exon 10 RNA is shown below with corresponding reading frame. (B) Western blotting shows the full-length human ataxin-3 protein with 84 Q repeats, the mouse ataxin-3 protein with 6 Qs, and the truncated ataxin-3 protein lacking the C terminus. Of the four AONs tested, AON 10.1 and 10.4 show the most efficient modification of the human ataxin-3 protein (Δ C terminus). Ctx = cortex, cb = cerebellum.

at 12 months of age.[45] This phenotype was associated with disturbances of endoplasmic reticulum-mediated unfolded protein response, possibly indicating the importance of ataxin-3 and VCP interaction in maintaining endoplasmic reticulum homeostasis.

A recent study by Liu and colleagues[46] testing several different oligonucleotide chemistries found that *ATXN3* exon 10 could be skipped with CAG-targeting oligonucleotides, and the ataxin-3 Δ C terminus protein was also observed in these experiments. Both mutant and wild-type ataxin-3 alleles will be targeted by the AON, as the AON is complementary to the canonical exon 10 sequence. Indeed, we observed a similar level of exon skipping for the mutant and wild-type allele in SCA3 patient-derived fibroblasts (Figures 1B and 1C). This observation underlines the importance of determining the protein function of ataxin-3 Δ C terminus in a neuronal context, as the proposed AON treatment may lead to high protein modification levels throughout the brain.

Apart from retaining wild-type ataxin-3 function, an important concern is that the ataxin-3 Δ C terminus protein should not result in a gain of toxic function. An ataxin-3 protein variant corresponding exactly to the Δ C terminus protein resulting from exon 10 skipping has been tested in a yeast model and rat cerebellar granule cells, where it was shown that this protein indeed did not aggregate, but in some tests showed a mild increase in toxicity compared to the wild-type ataxin-3 protein.[47,48] Whether these results can be extrapolated to the in vivo situation will have to be determined, but we did not observe overt signs of toxicity in the AON 10.4-treated mice expressing ataxin-3 Δ C terminus based on bodyweight and locomotor activity (Figures S3A and S3B). Additionally, no increase in cell death was observed in fibroblasts treated with AON 10.4 (Figure 1C), or U2OS cells transfected with GFP-ataxin-3 Δ C terminus (Figure 2B), indicating that ataxin-3 Δ C terminus is not toxic to these cells. Interestingly, a study investigating the transcript diversity of ataxin-3 in humans found two naturally occurring ataxin-3 isoforms generated by a stop codon after exon 9. Indeed, we observed an ataxin-3 protein of similar size to our Δ C terminus protein in untreated SCA3 animals (Figure 3B), perhaps indicating that this protein is also expressed in the transgenic mouse brain. If ataxin-3 Δ C terminus is indeed a naturally occurring isoform, this may suggest that the protein is likely not a toxic variant. To more comprehensively test whether ataxin-3 Δ C terminus is fully functional and not toxic to cells, it will be useful to express ataxin-3 Δ C terminus in ataxin-3 knockout mice in future studies.

The current study used the MJD84.2 mouse model.[24] This mouse model contains the full human *ATXN3* gene, including flanking regions and introns, which is essential when testing splice modulating AONs. Indeed, similar potency of the exon 10-targeting AONs was found when comparing patient-derived fibroblast results with the in vivo ICV injections. Repeated bolus injections of AON 10.4 were tolerated and led to increased levels of ataxin-3 protein modification. No significant reduction in the level of soluble mutant ataxin-3 with western blot analysis was observed (Figure 4C), however, a reduction in the amount of insoluble ataxin-3 was observed in the filter trap assay (Figure 4F).

We did not consistently observe strong nuclear accumulation of ataxin-3 in the deep cerebellar nuclei as previously reported in the MJD84.2 mice.[26] Though we were not able to detect clear aggregates in any brain regions, ataxin-3 nuclear localization was readily seen in the substantia nigra, in line with previous reports.[49] The molecular SCA3 phenotype of the mice was beneficially effected by AON treatment, with a reduction of insoluble ataxin-3 species observed in filter trap assays, and less nuclear ataxin-3 accumulation in the substantia nigra. The AONs were functionally active for at least 2.5 months, in line with previous studies using similar AON chemistries in spinal muscular atrophy mice reporting effects lasting for over 6 months after ICV injection.[50]

Clinical application of AONs currently seems promising. In clinical trials, intrathecal injection of 2′-O-methoxy-ethyl (MOE)-modified AONs in children with spinal muscular atrophy have shown AON half-lives in the cerebrospinal fluid (CSF) of 4–6 months, with good

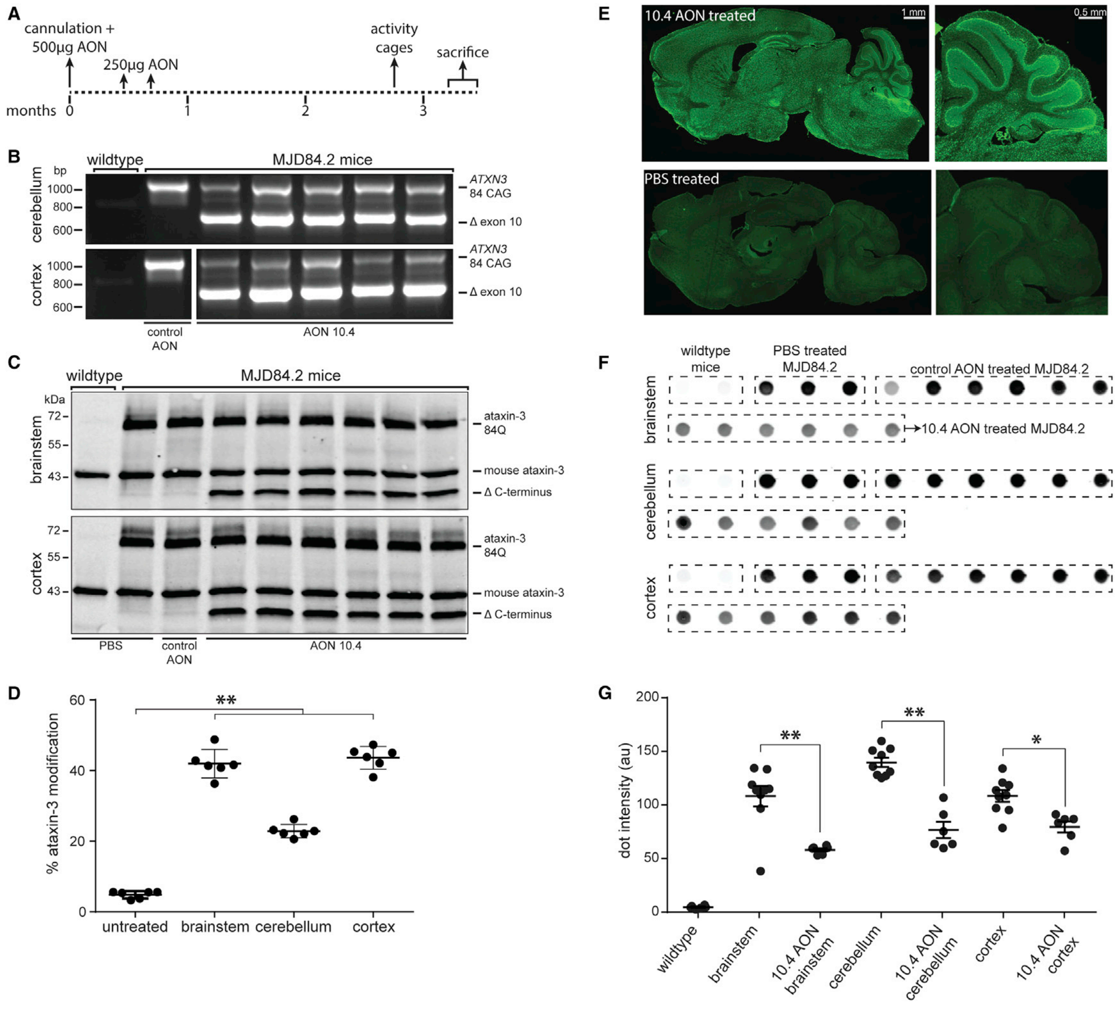

Figure 4. In Vivo Assessment of AON-Mediated Removal of the Ataxin-3 Polyglutamine Repeat in MJD84.2 Mice

(A) Schematic representation of experimental design. At ~2.5 months of age (time point 0), mice were cannulated and 500 µg of AON 10.4 was injected ICV. Additional 250 µg injections were performed at week 3 and 4 resulting in a total dose of 1 mg. Mice were sacrificed after 3.5 months. n = 6 per group. (B) RT-PCR specific for human *ATXN3* with primers flanking exon 10 shows full-length mutant ataxin-3 and ataxin-3 lacking exon 10 (Δ exon 10) in treated mice. (C) Modified ataxin-3 protein was seen for AON-treated transgenic mice in all tested brain regions: the brainstem and cortex are shown. Each lane represents one mouse. (D) Quantification of band intensity from (C) shows up to 40% ataxin-3 modification. Reported is modified ataxin-3 percentage of the sum of mutant and modified ataxin-3 band intensities. (E) Staining with an antibody against the phosphorothioate backbone shows that AONs are located throughout the entire mouse brain following ICV infusion. The upper panel shows a mouse sacrificed 3 months after injection with AON 10.4, and the lower panel depicts a PBS injected mouse. Higher magnification of the cerebellum shows good AON uptake in the Purkinje cells. (F) Filter retardation assay indicates that mice carrying the transgene show insoluble ataxin-3 in all tested brain regions when stained with 1H9 anti-ataxin-3 antibody. A reduction in insoluble ataxin-3 is seen in AON 10.4-treated animals compared to PBS- and control AON-treated animals. (G) Quantification of dot intensity from (F) reveals significant reduction in filter trapped insoluble ataxin-3 protein in AON 10.4-treated mice. *$p < 0.05$ and **$p < 0.01$.

tolerability and improvement in patients motor function.[51] This suggests that relatively infrequent dosing, such as intrathecal AON injections every 6 months, could be a feasible treatment regimen for SCA3. The removal of the polyQ repeat from ataxin-3 is likely to delay disease progression and restrict brain damage. In case of high efficacy of the treatment, it could even stop disease progression completely. Since the mouse model used here does not present with ataxic symptoms, we were unable to assess an effect of our AON treatment on reversal of the motor phenotype. However, studies in mice where expression of mutant ataxin-3 was halted during the early stage of the disease have shown that a reversal of the motor symptoms was possible.[52] A similar observation has been made when polyQ expanded Huntingtin protein was downregulated using AONs in a Huntington's disease mouse model.[53] This is a promising observation, but whether the same could be possible in humans is currently unknown. Because of the autosomal dominant nature of SCA3, patients can start therapy before onset of clinical symptoms, improving likelihood of a beneficial effect.

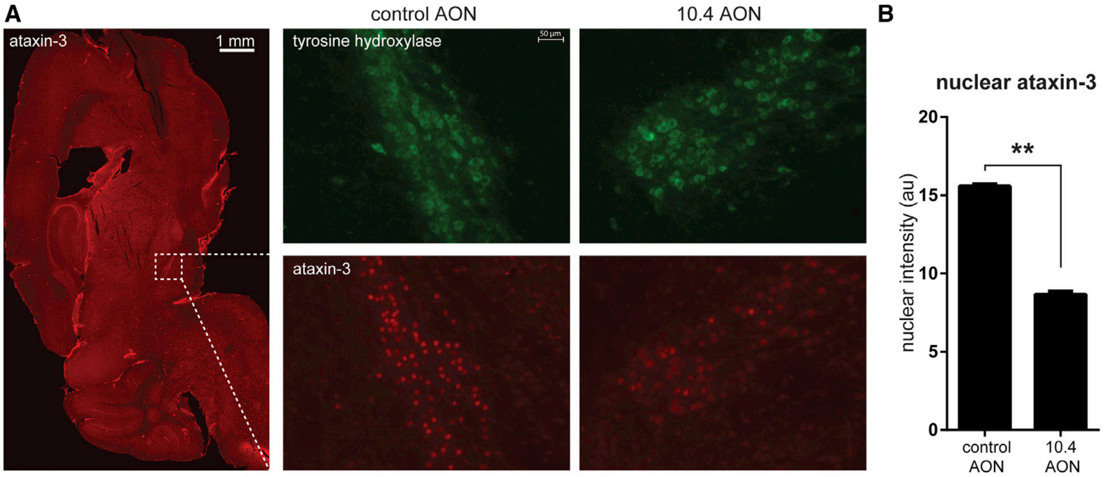

Figure 5. AON Treatment Reduces Nuclear Ataxin-3 Accumulation in Substantia Nigra of SCA3 Mice

(A) The nuclear accumulation of ataxin-3 was determined by staining with the 1H9 monoclonal antibody. Only cells of the substantia nigra expressing tyrosine hydroxylase were used in this analysis, as these showed the most intense nuclear ataxin-3 signal. (B) Quantification of staining intensity revealed that transgenic mice treated with 10.4 AON showed a decrease in nuclear ataxin-3 accumulation, likely due to the reduction in full-length mutant ataxin-3 levels. Analysis performed on four control AON versus three 10.4 AON-treated mice and four sections per mouse is shown. Mean + SEM, evaluated with Student's t test, $^{**}p < 0.01$.

MATERIALS AND METHODS

AON Design and Synthesis

Oligonucleotides targeting exon 10 (Table 1) of the human *ATXN3* transcript NCBI: NM_004993 were designed according to previously described guidelines.[54,55] AONs targeted predicted exonic splicing enhancer sites according to the Human Splicing Finder.[56] Specificity of the AONs was checked using BLAST analysis. The synthesis and purification of the oligonucleotides were performed as described previously.[57] AONs were fully modified with 2′-*O*-methoxyethylribose nucleotides and a phosphorothioate backbone. Cytosine residues were methylated to reduce immunostimulatory effects of the oligonucleotides in vivo.[58]

Cell Culture

SCA3 fibroblast (GM06153) cell lines were obtained from Coriell Cell Repositories (Camden) and maintained in minimal essential medium (MEM) (Gibco and Invitrogen), containing 15% fetal bovine serum (FBS) (Clontech), 1% Glutamax (Gibco), and 100 U/mL penicillin/streptomycin (Gibco).

Human U2OS 2-6-3 cells containing 200 copies of a LacO (256×)/TetO (96×)-containing cassette of ~4 Mbp[20] were cultured in DMEM (Gibco), supplemented with 10% FBS, 1% glutamax, and 100 U/mL penicillin/streptomycin. All cells were grown at 37°C and 5% CO_2.

Transfections

Transfections of AONs were performed as described previously.[40] Briefly, fibroblasts were replated the day before transfection. AONs were diluted to 200 nM in MEM without supplements containing 0.3% lipofectamine (Life Technologies). The cells were incubated with the transfection mixture for 4 hr, after which a three times volume of normal growth medium was added. Cells were harvested 1 day after transfection for RNA analysis or 2 days after transfection for protein analysis.

Plasmid transfections were performed similar to AON transfections using 1 to 1.5 µg of plasmid DNA (per 9.6 cm^2 well) in 0.6 mL MEM with 0.6% lipofectamine. Cells were imaged for GFP or mCherry protein expression the next day using a Leica DM-5500B fluorescent microscope (Leica Microsystems) at 63× magnification as previously described.[22]

Mice and Oligonucleotide Injections

MJD84.2 SCA3 mice[24] were obtained from Jackson laboratories, stock number 012705. All animal experiments were carried out in accordance with European Communities Council Directive 2010/63/EU and were approved by the Leiden University Animal Ethical Committee. Animals were housed singly after surgery in individually ventilated cages with a 12 hr light/dark cycle. Food and water were available ad libitum. Mice were bred with wild-type C57/BL6 mice to obtain wild-type or hemizygous SCA3 animals. Mice were genotyped using ear clip tissue material and a Phire Animal Tissue Direct PCR Kit (Thermo Fisher Scientific), using forward primer hAtxn3int10fw1 and reverse primer hAtxn3int10rev1 (see Table 2) targeting the human *ATXN3* transcript and mAtxn3int10fw1 and mAtxn3int10rev1 primers targeting mouse ataxin-3 as positive control for DNA isolation. Multiplex PCR was performed following manufacturer's instructions with an annealing temperature step of 61.5°C, 20 s of extension at 72°C, and a total of 35 PCR cycles.

For AON injections, a total of 34 male mice aged 2 to 2.5 months and weighing approximately 25 g were anesthetized using 1.5% isoflurane gas anesthesia and mounted on a Kopf stereotactic device (Kopf

Table 2. Primer Sequences

Target Gene	Primer Name	Application	Sequence (5′ to 3′)
ATXN3	hATXN3_FL_rev2	cDNA synthesis	TCCTACAACCGACGCATTGT
ATXN3	hATXN3_FL_Fw1	cloning	ATGGAGTCCATCTTCCACGA
ATXN3	hATXN3_FL_Rev	cloning	CGCATTGTTCCACTTTCCCA
ATXN3	hATXN3ex4Fw1	RT-PCR	GCCTTGAAAGTTTGGGGTTT
ATXN3	hATXN3ex11Rev1	RT-PCR	ACAGCTGCCTGAAGCATGTC
ATXN3	MJD_gen_fw1	genotyping PCR	ATACTTCACTTTTGAATGTTTCAGAC
ATXN3	MJD_gen_rev1	genotyping PCR	GAATGGTGAGCAGGCCTTAC
mATXN3	mAtxn3int10fw1	genotyping PCR	GCGTTGTTTTAACAGATATTCACG
mATXN3	mAtxn3int10rev1	genotyping PCR	TGTGAATGGACAGAAAGCAAA
ATNX3	hATXN3_ΔC14_fw	mutagenesis	GAAACAAGAAGGCTCACTTGCTCAACATTGCCTGAA
ATXN3	hATXN3_ΔC14_rev	mutagenesis	TTCAGGCAATGTTGAGCAAGTGAGCCTTCTTGTTTC

Instruments). Cannulas of 26 gauge and 3 mm in length (Plastics One) were implanted in the right lateral ventricle, according to the following coordinates: 0.2 mm posterior and 1.0 mm lateral to bregma and was lowered to a depth of 2.2 mm from the skull surface. The cannulas were subsequently fixed to the skull surface using dental cement and closed with a screw-on internal dummy cannula. AONs for in vivo injections were dissolved in sterile PBS without calcium or magnesium to a concentration of 50 μg/μL, as determined by UV spectrometry, and filtered using a 0.22 μm spin column filter. AONs or PBS were injected ICV through a 28 gauge needle placed in the guide cannula at a rate of 1 μL/min using a Hamilton syringe mounted in a syringe infusion pump (Stoelting). The first AON injection consisting of 500 μg in 10 μL was performed under anesthesia. Two additional ICV AON injections of 250 μg in 5 μL each were performed 2 and 3 weeks after surgery in freely moving mice, resulting in a total dose of 1 mg AON per mouse. Bodyweight was recorded every week post surgery. Mice were sacrificed 3.5 months after surgery. The left hemisphere was dissected and snap frozen in liquid nitrogen, the right hemisphere was placed in 4% paraformaldehyde and fixed overnight at 4°C. The fixed tissue was placed in 30% sucrose the next day and snap frozen in isopentane on dry ice.

RNA Isolation and RT-PCR

Cells were detached by trypsinization (Life Technologies) and subsequently spun down. RNA was collected from the cell pellets using the ReliaPrep RNA Cell Miniprep Kit (Promega) according to manufacturer's instructions. RNA from the mouse brain tissue was obtained from fresh frozen material by homogenization with a bullet blender BBX24 (Next Advance) for 3 min on setting 8, using pink beads (Next Advance), 500 μL TRIzol (Ambion and Thermo Fisher Scientific), and approximately 30 mg of tissue. After 5 min of incubation, 100 μL of chloroform was added and samples were spun down at 10,000 *g* for 15 min. The aqueous phase was removed and added to an equal volume of 70% ethanol. Further RNA purification was performed using the PureLink RNA Mini Kit (Thermo Fisher Scientific) in accordance with the manufacturer's protocol and using a 15 min DNase step. RNA was eluted in 80 μL nuclease free water.

For cDNA synthesis, 500 ng of RNA was used as input for the Transcriptor First Strand cDNA Synthesis Kit (Roche). The cDNA synthesis reaction was performed using oligoDT primers, or a gene-specific primer in the 3′ UTR region of human *ATXN3* for 45 min at 50°C and stopped for 5 min at 85°C, according to manufacturer's instructions. PCR was subsequently performed using primers in *ATXN3* exon 4 and 11 (see Table 2) with 1 μL cDNA as input. The PCR reaction contained 0.25 mM dNTPs, 1 U Faststart Taq DNA Polymerase (Roche), and 10 pmol forward and reverse primers (Eurogentec). PCR cycling was started with 4 min initial denaturation at 95°C, followed by a total of 36 cycles with 30 s of denaturation at 95°C, 30 s of annealing at 59°C, and 1 min extension at 72°C. At the end of the program, a final elongation step of 7 min at 72°C was used. PCR products were separated by electrophoresis on a 1.5% agarose gel containing 0.002% ethidium bromide. Bands of skipped products were excised from the gel, purified using a DNA extraction kit (Macherey-Nagel) according to manufacturer's instructions, and the sequence was obtained by Sanger sequencing (Macrogen).

Protein Isolation and Western Blotting

Protein from transfected cells was isolated by trypsinization and centrifugation of cells, after which, the pellet was dissolved in radioimmunoprecipitation assay (RIPA) buffer. Protein from ∼30 mg mouse brain tissue was isolated by homogenization with a bullet blender BBX24 for 3 min at intensity 8, in 500 μL RIPA buffer with 0.5 mm glass beads. Next, protein lysates were incubated in a head-over-head rotor at 4°C for 30 min. Protein concentration was determined using the bicinchoninic acid kit (Thermo Fisher Scientific), with BSA as a standard. Protein samples were separated using 10% SDS-PAGE with Laemmli sample buffer after boiling for 5 min at 100°C. Proteins were blotted onto a nitrocellulose membrane using the Trans Blot Turbo system (Bio-Rad) for 10 min at 1.3 A. Blocking of membranes was done with 5% low-fat milk powder in Tris-buffered saline (TBS) for 1 hr at room temperature. Membranes were stained using mouse anti-ataxin-3 1H9 (Abcam) at 1:5,000 dilution and rabbit antitubulin (Proteintech) 1:1,000 overnight at 4°C. Blots were washed and incubated for 1 hr with Odyssey secondary

antibodies, goat anti-mouse IRDye 680RD or goat anti-rabbit IRDye 800CW (LI-COR Biosciences) at a 1:5,000 dilution. Membranes were scanned using the Odyssey infrared imaging system (LI-COR Biosciences). Protein bands were quantified with the Odyssey software version 3.0 using the integrated intensity method. Percentage of protein modification was calculated based on band intensities as follows: modified ataxin-3 / (modified ataxin-3 + human ataxin-3 84Q) × 100.

Cryosectioning and Immunohistochemistry

Sectioning of paraformaldehyde-fixed mouse brains from four control AON- and three 10.4 AON-treated mice was performed using a Leica CM3050 cryostat. Sagittal sections of 25 μm thickness from the right hemisphere were immediately transferred as free-floating sections to PBS containing 0.02% sodium azide at room temperature. Sections were stored at 4°C until staining. Prior to staining, sections were washed three times in PBS with 0.2% Triton X-100 for 10 min and incubated with mouse on mouse (M.O.M.) mouse IgG blocking reagent (Vector Laboratories) for 1 hr. Sections were washed and incubated overnight at 4°C with primary antibodies diluted in M.O.M. protein concentrate diluent (Vector Laboratories). Primary antibodies used were: mouse anti-ataxin-3 1H9 1:1,000 (Abcam) and rabbit anti-tyrosine hydroxylase 1:500 (Santa Cruz Biotechnology). For assessment of AON distribution, a rabbit antibody binding the phosphorothioate backbone of AONs was used at 1:10,000 diluted in 1% normal goat serum. After washing, sections were incubated with secondary antibodies, goat anti-mouse Alexa Fluor 594, or goat anti-rabbit Alexa Fluor 488 (Life Technologies) at 1:500 dilution. Sections were mounted on superfrost plus coated microscope slides (Fisher Emergo), coverslipped using EverBrite Hardset Mounting Medium containing DAPI (Biotium), and cured overnight prior to fluorescent microscopic examination using a Leica DM-5500 using 63× magnification for assessment of ubiquitin binding or Keyence BIOREVO BZ-9000 fluorescent microscope for all other analyses at 10× magnification.

Image Analysis of Ataxin-3 Nuclear Intensity

Fluorescent images of substantia nigra were analyzed using ImageJ (version 1.48).[59] Images were converted to 8 bit and the substantia nigra was automatically selected using a region of interest based on positive tyrosine hydroxylase green fluorescence (threshold 25–254). Within this region, ataxin-3 nuclear staining was determined using the “Analyze Particles” function (circularity 0.15–1.00) based on red fluorescent staining (threshold 35–254). Background fluorescence (intensity 35) was subtracted, and the average fluorescence intensity per cell was subsequently used to represent intensity of nuclear ataxin-3. Identical analysis values were used to analyze all images. Between 400 and 1,300 individual cells were assessed for 10.4- and control AON-treated mice.

Dot-Blot Filter Retardation Assay

To detect aggregated ataxin-3 protein, mouse brain lysates in RIPA buffer were diluted in PBS to a final concentration of 0.15 μg/μL with 0.025% SDS. A total volume of 200 μL lysate was then passed through a cellulose acetate membrane of 0.2 μm (Whatman) mounted in a 96-well vacuum manifold. Three additional washes were performed using PBS to remove unbound protein. The membrane was blocked using 5% non-fat milk and subsequently stained for 3 hr with mouse anti-ataxin-3 1H9 1:5,000 (Abcam) and 1 hr with goat anti-mouse IRDye 800CW 1:10,000 (LI-COR Biosciences). Dots were quantified with the Odyssey software version 3.0 using the integrated intensity method.

Plasmids and Mutations

Plasmids for transfection were obtained as previously described.[22] In short, PCR products for cloning were generated with primers flanking the full-length *ATXN3* transcript (see Table 2) using AON transfected fibroblast cDNA as template. Full-length or exon 10-skipped products were gel extracted, purified, and ligated in the pGEM-T Easy Vector (Promega) using the 5′-A overhangs. Mutations of the UIMs and deletion of cysteine 14 was generated using the QuikChange II Site-Directed Mutagenesis Kit (Agilent Technologies) as described previously using primers containing the mutation (Table 2).[40] Expanded ataxin-3 with 71Q was obtained by gene synthesis (GenScript), a mixture of CAG and CAA codons was generated to improve stability during the cloning process. Constructs were then subcloned into the pAcGFP-C1 vector (Clontech) using notI digestion, resulting in an N-terminally GFP-tagged ataxin-3 protein expression. The mCherry-LacR-RNF8 construct has been described previously.[21] Purified ataxin-3 proteins were produced using the pET28a vector (Merck Millipore) and BL21 *E. coli* (New England Biolabs) as previously described.[22] All constructs were verified using Sanger sequencing.

Assessment of Ubiquitin Binding through RNF8 Chromatin Tethering

Ubiquitin binding assays were performed as previously described.[22] Briefly, human U2OS 2-6-3 cells with LacO repeats integrated in the genome[20] were grown on glass coverslips. Cells were transfected with both mCherry-LacR-RNF8[21] and GFP-ataxin-3 constructs. The cells were fixed in 4% paraformaldehyde the following day, and glass slides were mounted on microscope slides with EverBrite mounting medium containing DAPI (Biotium). Images were obtained for a minimum of 50 cells (two replicate transfections) positive for both RNF8 and ataxin-3 fluorescent signals. Due to the LacR fusion, the RNF8 protein construct localizes to the LacO repeat, resulting in local chromatin ubiquitination. Subsequently, ataxin-3 proteins localize to the ubiquitinated chromatin through their UIMs, resulting in colocalization with the mCherry-RNF8. The GFP intensity, thus representing ubiquitin binding, was quantified. By drawing a line region of interest across the mCherry-LacR-RNF8-marked array, the increase in GFP-ataxin-3 at the array was determined using the LAS AF Lite software (Leica Microsystems). The signal was background corrected by subtracting background GFP signal from the peak GFP intensity at the array.

Deubiquitination Assay

The in vitro deubiquitination assay was performed as previously described.[34]

1.2 μg HIS-ataxin-3 proteins were incubated with 0.5 μg K63-linked hexa-ubiquitin chains (Boston Biochem) in buffer containing 50 mM HEPES pH 8, 0.5 mM EDTA, and 1 mM DTT. Incubations were performed for 16 hr at 37°C. Reactions were stopped with 4× Laemmli sample buffer containing β-mercaptoethanol and by boiling the sample at 100°C for 5 min. Samples were run on SDS-PAGE using 10% TGX gels (Bio-Rad). After western blotting, membranes were probed with anti-ataxin-3 1H9 (1:5,000) and anti-ubiquitin 1:1,000 (UG9511) (Enzo Life Sciences).

Statistical Analyses

The percentage of ataxin-3 protein modification, filter trap assay dot intensity, and ataxin-3 ubiquitin binding assays were analyzed with one-way ANOVA using Tukey's post hoc multiple comparisons test. Raw intensity values obtained from Oyssey application software "integrated intensity quantification" method were used for analyses of filter trap assays and western blots. Western blot protein modification is reported as percentage modified ataxin-3 of the sum of all human ataxin-3 bands. Intensity of nuclear ataxin-3 accumulation in substantia nigra was compared using unpaired Student's t test. Statistical analyses were performed using GraphPad Prism software version 6.02. p values < 0.05 were considered statistically significant.

SUPPLEMENTAL INFORMATION

Supplemental Information includes three figures and can be found with this article online at http://dx.doi.org/10.1016/j.omtn.2017.06.019.

AUTHOR CONTRIBUTIONS

Research conception and experiment design: W.M.C.v.R.-M., L.J.A.T., F.R., and H.v.A. Experiments and manuscript writing: L.J.A.T. Revision of manuscript: W.M.C.v.R.-M., H.v.A., and F.R.

ACKNOWLEDGMENTS

The authors want to thank Maarten Schenke, Barry Pepers, Martijn Luijsterburg, and Melvin Evers for advice on experimental setup and Maurice Overzier and Wouter Wiegant for practical assistance. The authors are grateful to Susan Janicki and Roger Greenberg for providing U2OS 2-6-3 cells. This work was financially supported by ZonMw (40-41900-98-018) and Hersenstichting/Brugling Fund (BG2013-03) to W.M.C.v.R.-M.

REFERENCES

1. Rüb, U., Schöls, L., Paulson, H., Auburger, G., Kermer, P., Jen, J.C., Seidel, K., Korf, H.W., and Deller, T. (2013). Clinical features, neurogenetics and neuropathology of the polyglutamine spinocerebellar ataxias type 1, 2, 3, 6 and 7. Prog. Neurobiol. *104*, 38–66.

2. Riess, O., Rüb, U., Pastore, A., Bauer, P., and Schöls, L. (2008). SCA3: neurological features, pathogenesis and animal models. Cerebellum *7*, 125–137.

3. Orr, H.T., and Zoghbi, H.Y. (2007). Trinucleotide repeat disorders. Annu. Rev. Neurosci. *30*, 575–621.

4. Kawaguchi, Y., Okamoto, T., Taniwaki, M., Aizawa, M., Inoue, M., Katayama, S., Kawakami, H., Nakamura, S., Nishimura, M., Akiguchi, I., et al. (1994). CAG expansions in a novel gene for Machado-Joseph disease at chromosome 14q32.1. Nat. Genet. *8*, 221–228.

5. Cummings, C.J., and Zoghbi, H.Y. (2000). Trinucleotide repeats: mechanisms and pathophysiology. Annu. Rev. Genomics Hum. Genet. *1*, 281–328.

6. Matos, C.A., de Macedo-Ribeiro, S., and Carvalho, A.L. (2011). Polyglutamine diseases: the special case of ataxin-3 and Machado-Joseph disease. Prog. Neurobiol. *95*, 26–48.

7. Bettencourt, C., Raposo, M., Ros, R., Montiel, R., Bruges-Armas, J., and Lima, M. (2013). Transcript diversity of Machado-Joseph disease gene (ATXN3) is not directly determined by SNPs in exonic or flanking intronic regions. J. Mol. Neurosci. *49*, 539–543.

8. Harris, G.M., Dodelzon, K., Gong, L., Gonzalez-Alegre, P., and Paulson, H.L. (2010). Splice isoforms of the polyglutamine disease protein ataxin-3 exhibit similar enzymatic yet different aggregation properties. PLoS ONE *5*, e13695.

9. Schmidt, T., Landwehrmeyer, G.B., Schmitt, I., Trottier, Y., Auburger, G., Laccone, F., Klockgether, T., Völpel, M., Epplen, J.T., Schöls, L., and Riess, O. (1998). An isoform of ataxin-3 accumulates in the nucleus of neuronal cells in affected brain regions of SCA3 patients. Brain Pathol. *8*, 669–679.

10. Chai, Y., Berke, S.S., Cohen, R.E., and Paulson, H.L. (2004). Poly-ubiquitin binding by the polyglutamine disease protein ataxin-3 links its normal function to protein surveillance pathways. J. Biol. Chem. *279*, 3605–3611.

11. Mao, Y., Senic-Matuglia, F., Di Fiore, P.P., Polo, S., Hodsdon, M.E., and De Camilli, P. (2005). Deubiquitinating function of ataxin-3: insights from the solution structure of the Josephin domain. Proc. Natl. Acad. Sci. USA *102*, 12700–12705.

12. Winborn, B.J., Travis, S.M., Todi, S.V., Scaglione, K.M., Xu, P., Williams, A.J., Cohen, R.E., Peng, J., and Paulson, H.L. (2008). The deubiquitinating enzyme ataxin-3, a polyglutamine disease protein, edits Lys63 linkages in mixed linkage ubiquitin chains. J. Biol. Chem. *283*, 26436–26443.

13. Alves, S., Nascimento-Ferreira, I., Dufour, N., Hassig, R., Auregan, G., Nóbrega, C., Brouillet, E., Hantraye, P., Pedroso de Lima, M.C., Déglon, N., and de Almeida, L.P. (2010). Silencing ataxin-3 mitigates degeneration in a rat model of Machado-Joseph disease: no role for wild-type ataxin-3? Hum. Mol. Genet. *19*, 2380–2394.

14. Alves, S., Nascimento-Ferreira, I., Auregan, G., Hassig, R., Dufour, N., Brouillet, E., Pedroso de Lima, M.C., Hantraye, P., Pereira de Almeida, L., and Déglon, N. (2008). Allele-specific RNA silencing of mutant ataxin-3 mediates neuroprotection in a rat model of Machado-Joseph disease. PLoS ONE *3*, e3341.

15. Conceição, M., Mendonça, L., Nóbrega, C., Gomes, C., Costa, P., Hirai, H., Moreira, J.N., Lima, M.C., Manjunath, N., and Pereira de Almeida, L. (2016). Intravenous administration of brain-targeted stable nucleic acid lipid particles alleviates Machado-Joseph disease neurological phenotype. Biomaterials *82*, 124–137.

16. Nóbrega, C., Nascimento-Ferreira, I., Onofre, I., Albuquerque, D., Hirai, H., Déglon, N., and de Almeida, L.P. (2013). Silencing mutant ataxin-3 rescues motor deficits and neuropathology in Machado-Joseph disease transgenic mice. PLoS ONE *8*, e52396.

17. Evers, M.M., Toonen, L.J., and van Roon-Mom, W.M. (2015). Antisense oligonucleotides in therapy for neurodegenerative disorders. Adv. Drug Deliv. Rev. *87*, 90–103.

18. Gaspar, C., Lopes-Cendes, I., Hayes, S., Goto, J., Arvidsson, K., Dias, A., Silveira, I., Maciel, P., Coutinho, P., Lima, M., et al. (2001). Ancestral origins of the Machado-Joseph disease mutation: a worldwide haplotype study. Am. J. Hum. Genet. *68*, 523–528.

19. Michlewski, G., and Krzyzosiak, W.J. (2004). Molecular architecture of CAG repeats in human disease related transcripts. J. Mol. Biol. *340*, 665–679.

20. Janicki, S.M., Tsukamoto, T., Salghetti, S.E., Tansey, W.P., Sachidanandam, R., Prasanth, K.V., Ried, T., Shav-Tal, Y., Bertrand, E., Singer, R.H., and Spector, D.L. (2004). From silencing to gene expression: real-time analysis in single cells. Cell *116*, 683–698.

21. Luijsterburg, M.S., Acs, K., Ackermann, L., Wiegant, W.W., Bekker-Jensen, S., Larsen, D.H., Khanna, K.K., van Attikum, H., Mailand, N., and Dantuma, N.P. (2012). A new non-catalytic role for ubiquitin ligase RNF8 in unfolding higher-order chromatin structure. EMBO J. *31*, 2511–2527.

22. Toonen, L.J., Schmidt, I., Luijsterburg, M.S., van Attikum, H., and van Roon-Mom, W.M. (2016). Antisense oligonucleotide-mediated exon skipping as a strategy to reduce proteolytic cleavage of ataxin-3. Sci. Rep. *6*, 35200.

23. Burnett, B., Li, F., and Pittman, R.N. (2003). The polyglutamine neurodegenerative protein ataxin-3 binds polyubiquitylated proteins and has ubiquitin protease activity. Hum. Mol. Genet. *12*, 3195–3205.

24. Cemal, C.K., Carroll, C.J., Lawrence, L., Lowrie, M.B., Ruddle, P., Al-Mahdawi, S., King, R.H., Pook, M.A., Huxley, C., and Chamberlain, S. (2002). YAC transgenic mice carrying pathological alleles of the MJD1 locus exhibit a mild and slowly progressive cerebellar deficit. Hum. Mol. Genet. *11*, 1075–1094.

25. Costa Mdo, C., Luna-Cancalon, K., Fischer, S., Ashraf, N.S., Ouyang, M., Dharia, R.M., Martin-Fishman, L., Yang, Y., Shakkottai, V.G., Davidson, B.L., et al. (2013). Toward RNAi therapy for the polyglutamine disease Machado-Joseph disease. Mol. Ther. *21*, 1898–1908.

26. Rodriguez-Lebron, E., Costa Mdo, C., Luna-Cancalon, K., Peron, T.M., Fischer, S., Boudreau, R.L., Davidson, B.L., and Paulson, H.L. (2013). Silencing mutant ATXN3 expression resolves molecular phenotypes in SCA3 transgenic mice. Mol. Ther. *21*, 1909–1918.

27. Chen, S., Ferrone, F.A., and Wetzel, R. (2002). Huntington's disease age-of-onset linked to polyglutamine aggregation nucleation. Proc. Natl. Acad. Sci. USA *99*, 11884–11889.

28. Holmes, W.M., Klaips, C.L., and Serio, T.R. (2014). Defining the limits: protein aggregation and toxicity in vivo. Crit. Rev. Biochem. Mol. Biol. *49*, 294–303.

29. Koch, P., Breuer, P., Peitz, M., Jungverdorben, J., Kesavan, J., Poppe, D., Doerr, J., Ladewig, J., Mertens, J., Tüting, T., et al. (2011). Excitation-induced ataxin-3 aggregation in neurons from patients with Machado-Joseph disease. Nature *480*, 543–546.

30. Wanker, E.E., Scherzinger, E., Heiser, V., Sittler, A., Eickhoff, H., and Lehrach, H. (1999). Membrane filter assay for detection of amyloid-like polyglutamine-containing protein aggregates. Methods Enzymol. *309*, 375–386.

31. Bichelmeier, U., Schmidt, T., Hübener, J., Boy, J., Rüttiger, L., Häbig, K., Poths, S., Bonin, M., Knipper, M., Schmidt, W.J., et al. (2007). Nuclear localization of ataxin-3 is required for the manifestation of symptoms in SCA3: in vivo evidence. J. Neurosci. *27*, 7418–7428.

32. Evers, M.M., Toonen, L.J., and van Roon-Mom, W.M. (2014). Ataxin-3 protein and RNA toxicity in spinocerebellar ataxia type 3: current insights and emerging therapeutic strategies. Mol. Neurobiol. *49*, 1513–1531.

33. Evert, B.O., Araujo, J., Vieira-Saecker, A.M., de Vos, R.A., Harendza, S., Klockgether, T., and Wüllner, U. (2006). Ataxin-3 represses transcription via chromatin binding, interaction with histone deacetylase 3, and histone deacetylation. J. Neurosci. *26*, 11474–11486.

34. Pfeiffer, A., Luijsterburg, M.S., Acs, K., Wiegant, W.W., Helfricht, A., Herzog, L.K., Minoia, M., Böttcher, C., Salomons, F.A., van Attikum, H., and Dantuma, N.P. (2017). Ataxin-3 consolidates the MDC1-dependent DNA double-strand break response by counteracting the SUMO-targeted ubiquitin ligase RNF4. Embo J. *36*, 1066–1083.

35. Moore, L.R., Rajpal, G., Dillingham, I.T., Qutob, M., Blumenstein, K.G., Gattis, D., Hung, G., Kordasiewicz, H.B., Paulson, H.L., and McLoughlin, H.S. (2017). Evaluation of antisense oligonucleotides targeting ATXN3 in SCA3 mouse models. Mol. Ther. Nucleic Acids *7*, 200–210.

36. Schmitt, I., Linden, M., Khazneh, H., Evert, B.O., Breuer, P., Klockgether, T., and Wuellner, U. (2007). Inactivation of the mouse Atxn3 (ataxin-3) gene increases protein ubiquitination. Biochem. Biophys. Res. Commun. *362*, 734–739.

37. Switonski, P.M., Fiszer, A., Kazmierska, K., Kurpisz, M., Krzyzosiak, W.J., and Figiel, M. (2011). Mouse ataxin-3 functional knock-out model. Neuromolecular Med. *13*, 54–65.

38. Boeddrich, A., Gaumer, S., Haacke, A., Tzvetkov, N., Albrecht, M., Evert, B.O., Müller, E.C., Lurz, R., Breuer, P., Schugardt, N., et al. (2006). An arginine/lysine-rich motif is crucial for VCP/p97-mediated modulation of ataxin-3 fibrillogenesis. EMBO J. *25*, 1547–1558.

39. Nicastro, G., Todi, S.V., Karaca, E., Bonvin, A.M., Paulson, H.L., and Pastore, A. (2010). Understanding the role of the Josephin domain in the PolyUb binding and cleavage properties of ataxin-3. PLoS ONE *5*, e12430.

40. Evers, M.M., Tran, H.D., Zalachoras, I., Pepers, B.A., Meijer, O.C., den Dunnen, J.T., van Ommen, G.J., Aartsma-Rus, A., and van Roon-Mom, W.M. (2013). Ataxin-3 protein modification as a treatment strategy for spinocerebellar ataxia type 3: removal of the CAG containing exon. Neurobiol. Dis. *58*, 49–56.

41. Donaldson, K.M., Li, W., Ching, K.A., Batalov, S., Tsai, C.C., and Joazeiro, C.A. (2003). Ubiquitin-mediated sequestration of normal cellular proteins into polyglutamine aggregates. Proc. Natl. Acad. Sci. USA *100*, 8892–8897.

42. Tzvetkov, N., and Breuer, P. (2007). Josephin domain-containing proteins from a variety of species are active de-ubiquitination enzymes. Biol. Chem. *388*, 973–978.

43. Laço, M.N., Cortes, L., Travis, S.M., Paulson, H.L., and Rego, A.C. (2012). Valosin-containing protein (VCP/p97) is an activator of wild-type ataxin-3. PLoS ONE *7*, e43563.

44. Zhong, X., and Pittman, R.N. (2006). Ataxin-3 binds VCP/p97 and regulates retrotranslocation of ERAD substrates. Hum. Mol. Genet. *15*, 2409–2420.

45. Hübener, J., Vauti, F., Funke, C., Wolburg, H., Ye, Y., Schmidt, T., Wolburg-Buchholz, K., Schmitt, I., Gardyan, A., Driessen, S., et al. (2011). N-terminal ataxin-3 causes neurological symptoms with inclusions, endoplasmic reticulum stress and ribosomal dislocation. Brain *134*, 1925–1942.

46. Liu, J., Yu, D., Aiba, Y., Pendergraff, H., Swayze, E.E., Lima, W.F., Hu, J., Prakash, T.P., and Corey, D.R. (2013). ss-siRNAs allele selectively inhibit ataxin-3 expression: multiple mechanisms for an alternative gene silencing strategy. Nucleic Acids Res. *41*, 9570–9583.

47. Bonanomi, M., Visentin, C., Invernizzi, G., Tortora, P., and Regonesi, M.E. (2015). The toxic effects of pathogenic ataxin-3 variants in a yeast cellular model. PLoS ONE *10*, e0129727.

48. Pellistri, F., Bucciantini, M., Invernizzi, G., Gatta, E., Penco, A., Frana, A.M., Nosi, D., Relini, A., Regonesi, M.E., Gliozzi, A., et al. (2013). Different ataxin-3 amyloid aggregates induce intracellular Ca(2+) deregulation by different mechanisms in cerebellar granule cells. Biochim. Biophys. Acta *1833*, 3155–3165.

49. Chen, X., Tang, T.S., Tu, H., Nelson, O., Pook, M., Hammer, R., Nukina, N., and Bezprozvanny, I. (2008). Deranged calcium signaling and neurodegeneration in spinocerebellar ataxia type 3. J. Neurosci. *28*, 12713–12724.

50. Rigo, F., Chun, S.J., Norris, D.A., Hung, G., Lee, S., Matson, J., Fey, R.A., Gaus, H., Hua, Y., Grundy, J.S., et al. (2014). Pharmacology of a central nervous system delivered 2′-O-methoxyethyl-modified survival of motor neuron splicing oligonucleotide in mice and nonhuman primates. J. Pharmacol. Exp. Ther. *350*, 46–55.

51. Chiriboga, C.A., Swoboda, K.J., Darras, B.T., Iannaccone, S.T., Montes, J., De Vivo, D.C., Norris, D.A., Bennett, C.F., and Bishop, K.M. (2016). Results from a phase 1 study of nusinersen (ISIS-SMN(Rx)) in children with spinal muscular atrophy. Neurology *86*, 890–897.

52. Boy, J., Schmidt, T., Wolburg, H., Mack, A., Nuber, S., Böttcher, M., Schmitt, I., Holzmann, C., Zimmermann, F., Servadio, A., and Riess, O. (2009). Reversibility of symptoms in a conditional mouse model of spinocerebellar ataxia type 3. Hum. Mol. Genet. *18*, 4282–4295.

53. Kordasiewicz, H.B., Stanek, L.M., Wancewicz, E.V., Mazur, C., McAlonis, M.M., Pytel, K.A., Artates, J.W., Weiss, A., Cheng, S.H., Shihabuddin, L.S., et al. (2012). Sustained therapeutic reversal of Huntington's disease by transient repression of huntingtin synthesis. Neuron *74*, 1031–1044.

54. Aartsma-Rus, A. (2012). Overview on AON design. Methods Mol. Biol. *867*, 117–129.

55. Aartsma-Rus, A., van Vliet, L., Hirschi, M., Janson, A.A., Heemskerk, H., de Winter, C.L., de Kimpe, S., van Deutekom, J.C.T., Hoen, P.A.C.'t, and van Ommen, G.-J.B. (2009). Guidelines for antisense oligonucleotide design and insight into splice-modulating mechanisms. Mol. Ther. *17*, 548–553.

56. Desmet, F.O., Hamroun, D., Lalande, M., Collod-Béroud, G., Claustres, M., and Béroud, C. (2009). Human splicing finder: an online bioinformatics tool to predict splicing signals. Nucleic Acids Res. *37*, e67.

57. Swayze, E.E., Siwkowski, A.M., Wancewicz, E.V., Migawa, M.T., Wyrzykiewicz, T.K., Hung, G., Monia, B.P., and Bennett, C.F. (2007). Antisense oligonucleotides containing locked nucleic acid improve potency but cause significant hepatotoxicity in animals. Nucleic Acids Res. *35*, 687–700.

58. Bauer, S., Kirschning, C.J., Häcker, H., Redecke, V., Hausmann, S., Akira, S., Wagner, H., and Lipford, G.B. (2001). Human TLR9 confers responsiveness to bacterial DNA via species-specific CpG motif recognition. Proc. Natl. Acad. Sci. USA *98*, 9237–9242.

59. Schneider, C.A., Rasband, W.S., and Eliceiri, K.W. (2012). NIH Image to ImageJ: 25 years of image analysis. Nat. Methods *9*, 671–675.

OMTN, Volume *8*

Supplemental Information

Antisense Oligonucleotide-Mediated Removal of the Polyglutamine Repeat in Spinocerebellar Ataxia Type 3 Mice

Lodewijk J.A. Toonen, Frank Rigo, Haico van Attikum, and Willeke M.C. van Roon-Mom

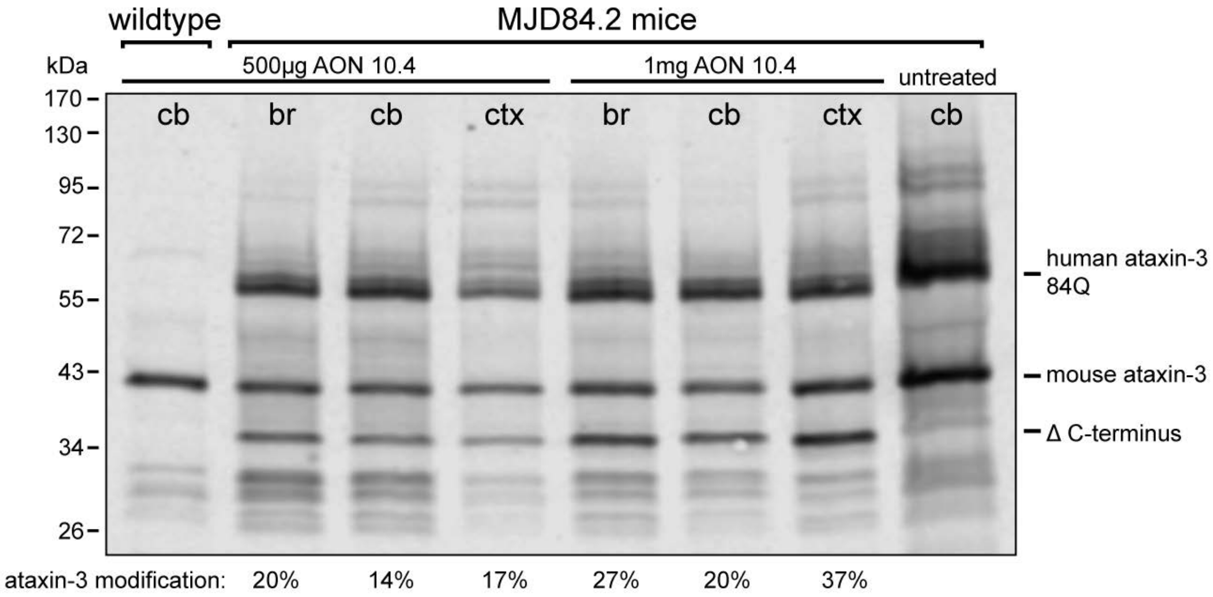

Supplementary Figure 1: Comparison of 500 µg and 1 mg AON 10.4 ICV bolus. AON 10.4 was tested *in vivo* by ICV injection in two mice to determine dose response. 500 µg AON was injected at 2.5 months of age, after which one of the mice was injected with a second 500 µg bolus 2 weeks later. Both mice were sacrificed 2 weeks after the last injection. Westernblot analysis and staining with 1H9 antibody shows ataxin-3 Δ C-terminus appearing in the three brainregions tested of the treated MJD84.2 mice. A clear increase in protein modifciation is seen with the 1 mg dose compared to the 500 µg dose. Cb = cerebellum, br = brainstem, ctx = cortex.

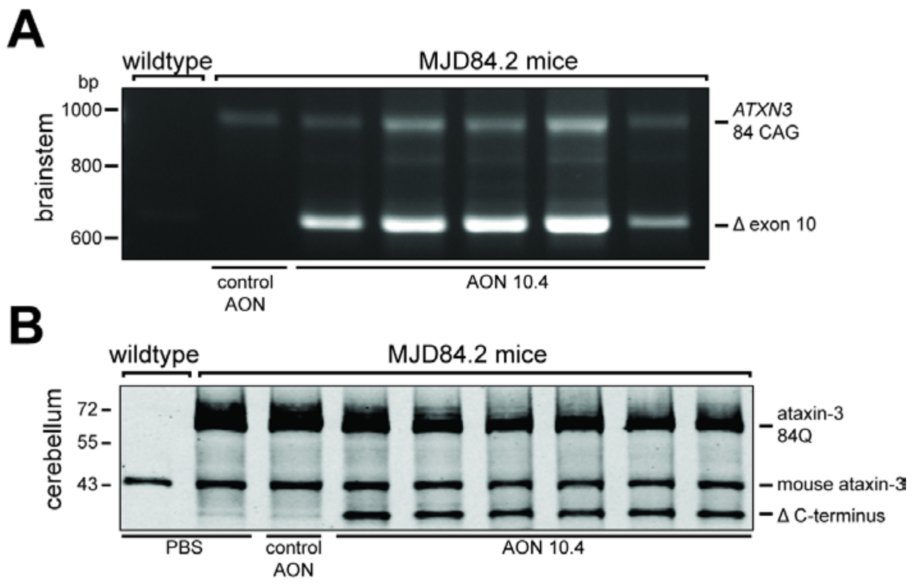

Supplementary Figure 2: In vivo assessment of exon skipping at RNA and protein level. Mice were treated with a total of 1 mg AON 10.4 or control AON, and sacrificed ~3.5 monhs after last injection. Results are obtained from mice depicted in figure 3. (**A**) RT-PCR with primers for human *ATXN3* show skipping of exon 10 in brainstem of AON 10.4 treated mice. (**B)** Modified ataxin-3 protein (Δ C-terminus) was observed in cerebellum of mice treated with AON 10.4.

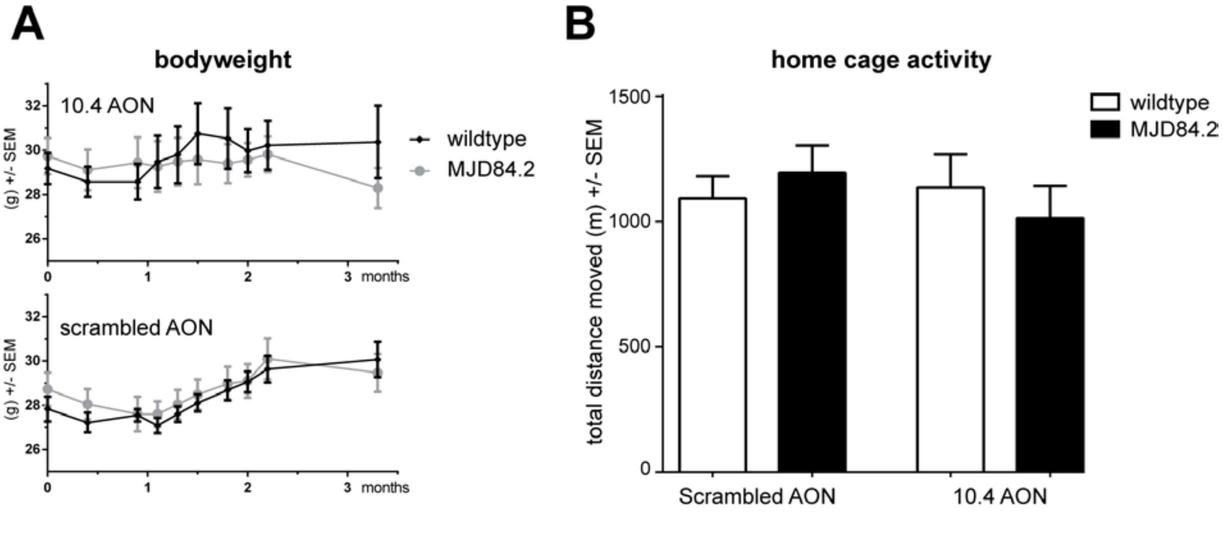

Supplementary Figure 3: MJD84.2 mouse does not present obvious ataxic phenotype at 5 months of age. (A) No significant difference in bodyweight between wildtype and transgenic mice was seen, or between scrambled and AON10.4 treated mice of both genotypes. (**B**) Wildtype and hemizygous MJD84.2 mice were tested for motor performance in a home cage activity system at around 5 months of age. Scrambled AON: 5 wildtype vs 5 SCA3 mice. 10.4 AON: 4 wildtype vs 6 SCA3 mice.
